# Supplementary material for: Mechanisms of Differential Signal Transduction by IFNLR1 Variants
Source: bioRxiv. 2025 Oct 4:2025.10.03.677101. Preprint. [Version 1] doi: 10.1101/2025.10.03.677101 (PMC12621819; doi:10.1101/2025.10.03.677101)
Supplement: Supplement 2 [file NIHPP2025.10.03.677101v1-supplement-2.pdf]

bioRxiv preprint doi: https://doi.org/10.1101/2025.10.03.677101; this version posted October 4, 2025. The copyright holder for this preprint (which was not certified by peer review) is the author/funder, who has granted bioRxiv a license to display the preprint in perpetuity. It is made available under aCC-BY 4.0 International license.

Supplemental Table 1. Primer-probe sets used for gene expression analyses

bioRxiv preprint doi: https://doi.org/10.1101/2025.10.03.677101; this version posted October 4, 2025. The copyright holder for this preprint (which was not certified by peer review) is the author/funder, who has granted bioRxiv a license to display the preprint in perpetuity. It is made available under aCC-BY 4.0 International license.

Gene	Assay ID	Vendor
CXCL10	Hs0017042_m1	ThermoFisher
GAPDH	Hs02786624_g1	ThermoFisher
IFITM1	Hs06057129_s1	ThermoFisher
IFITM3	Hs00705137_s1	ThermoFisher
IFNLR1 isoform 1	Hs00417120_m1	ThermoFisher
IFNLR1 isoform 2	Hs00906642_m1	ThermoFisher
IFNLR1 isoform 3	Hs00906643_m1	ThermoFisher
MX1	Hs00895608_m1	ThermoFisher
RSAD2 (VIPERIN)	Hs00369813_m1	ThermoFisher

Supplemental Figure 1

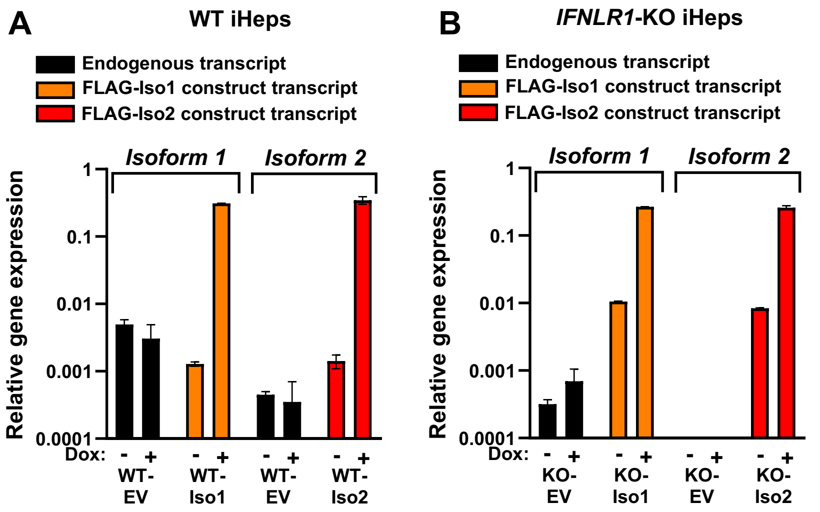

Supplemental Figure 1. Relative expression of endogenous *IFNLR1* and FLAG-Iso1 and -Iso2 transcripts in (**A**) wild type (WT) and (**B**) *IFNLR1*-knock out (KO) iHeps with or without doxycycline induction, determined by qRT-PCR. Biological replicates were assayed in technical duplicate and mean ± SEM is shown relative to *GAPDH*.

Supplemental Figure 2

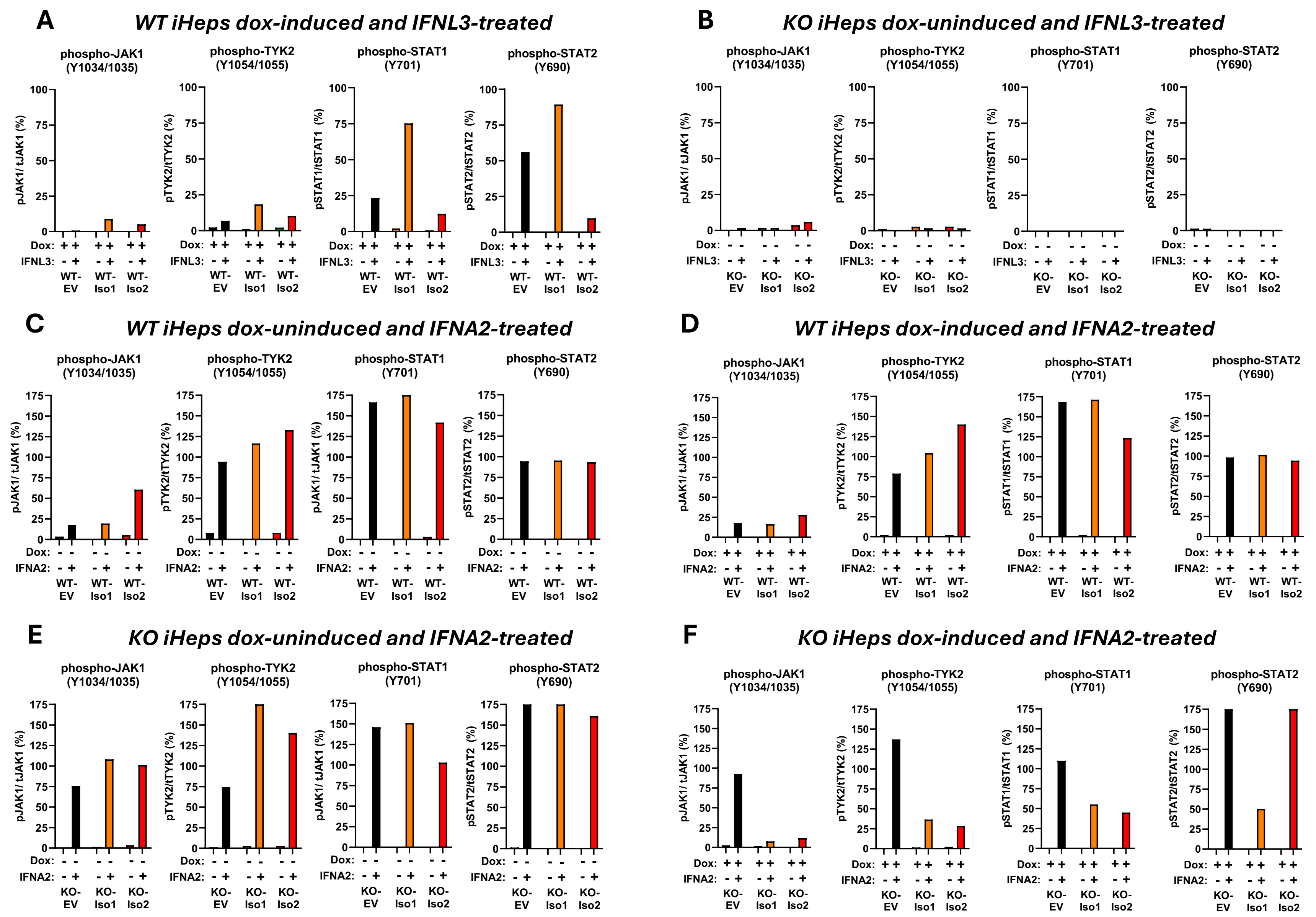

Supplemental Figure 2. Percentage of phosphorylated to total protein in WT and KO iHep western blots shown in Fig. 4 for (**A**) dox-induced WT iHeps +/- IFNL3, (**B**) dox-uninduced *IFNLR1*-KO iHeps +/- IFNL3, (**C**) dox-uninduced WT iHeps +/- IFNA2 (**D**) dox-induced WT iHeps +/- IFNA2, (**E**) dox-uninduced *IFNLR1*-KO iHeps +/- IFNA2 and (**F**) dox-induced *IFNLR1*-KO iHeps +/- IFNA2 for 15min. Integrated band intensity was determined in ImageJ.

Supplemental Figure 3

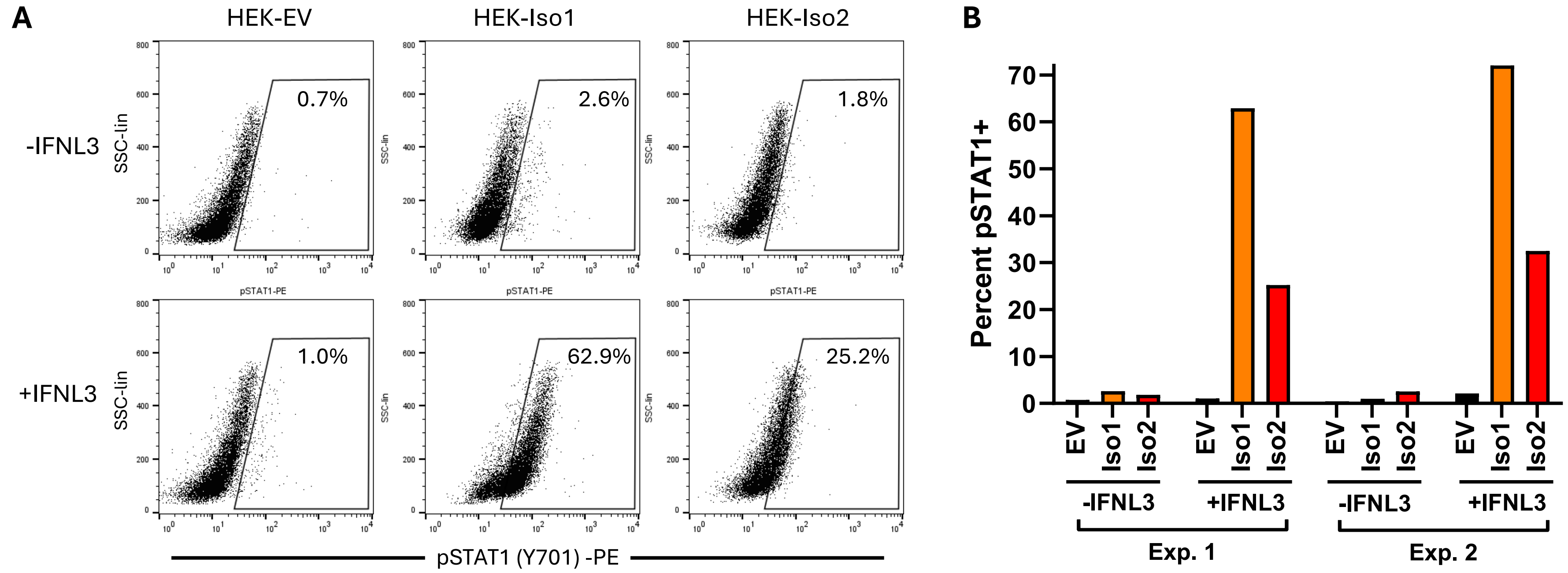

Supplemental Figure 3. IFNLR1 variant 1 drives greater IFNL3-induced activation of STAT1 compared to variant 2. (**A**) Representative flow cytometry scatterplots depicting HEK293T cells incubated with or without IFNL3 for 15min then labeled for pSTAT1. The proportion of each population with positive signal is indicated within each scatterplot and summary results from two independent experiments are shown in **(B).**

Supplemental Figure 4

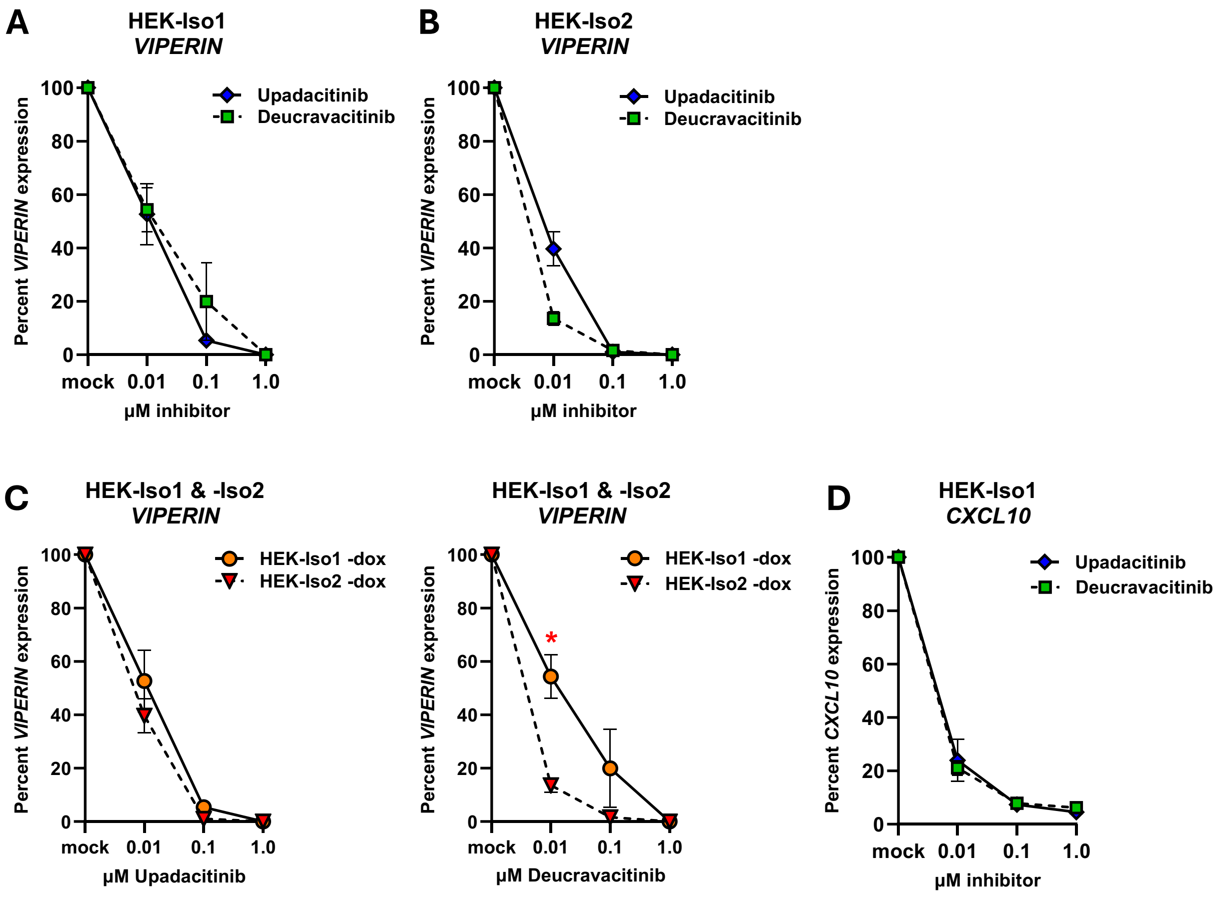

Supplemental Figure 4. Percent antiviral (*VIPERIN*) and proinflammatory (*CXCL10*) gene expression in HEK293T cells pre-treated with JAK1- (Upadacitinib; IC_{50} 47nM) or TYK2-(Deucravacitinib; IC_{50} 0.2nM) inhibitors prior to IFNL3 stimulation. *VIPERIN* expression in dox-uninduced (**A**) HEK-Iso1 and (**B**) HEK-Iso2 cells. (**C**) Comparison of percent *VIPERIN* expression between HEK-Iso1 and –Iso2 cell lines. (**D**) Percent expression of *CXCL10* in HEK-Iso1 cells. Biological replicates were assayed in technical duplicate and mean ± SEM is shown relative to *GAPDH*. Percent gene expression was calculated relative to respective mock treated samples. *p≤ 0.05 by Student's t-test. Data are representative of two independent experiments.

Supplemental Figure 5

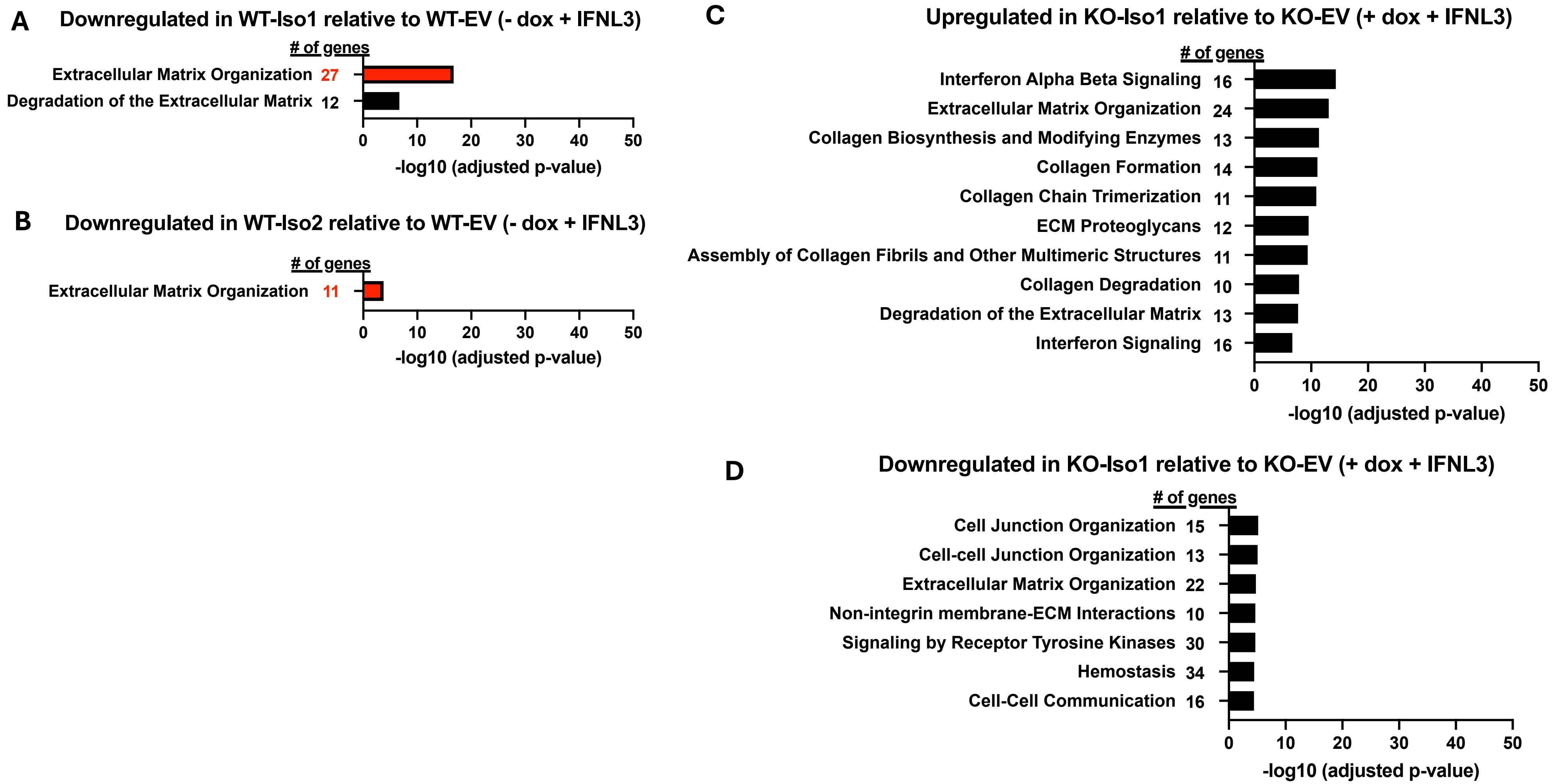

Supplemental Figure 5. IFNLR1 variants support differential gene expression. Top pathways down-regulated by IFNL3 treatment in dox-uninduced WT-Iso1 (**A**) or WT-Iso2 (**B**) iHeps relative to similarly treated WT-EV iHeps. Only pathways with at least 10 dataset genes represented in the pathway are shown. Pathways represented in both datasets are indicated in red, and the number of genes from the dataset represented in each individual pathway are indicated. Top up- (**C**) and down-regulated (**D**) pathways in dox-induced, IFNL3-treated KO-Iso1 iHeps relative to similarly treated KO–EV iHeps. No up or down-regulated pathways met the selected significance criteria (see Methods) for KO-Iso2 iHeps.
